# Crosstalk Between Macroautophagy and Chaperone-Mediated Autophagy: Implications for the Treatment of Neurological Diseases

**DOI:** 10.1007/s12035-014-8933-0

**Published:** 2014-10-21

**Authors:** Haijian Wu, Sheng Chen, Al-Baadani Ammar, Jie Xu, Qun Wu, Kum Pan, Jianmin Zhang, Yuan Hong

**Affiliations:** 1 Department of Neurosurgery, Second Affiliated Hospital, School of Medicine, Zhejiang University, Hangzhou, China; 2 Department of Neurological Surgery, Weill Cornell Medical College, New York, USA

**Keywords:** Macroautophagy, Chaperone-mediated autophagy, Interplay, Cell biology, Neurological disease

## Abstract

Macroautophagy and chaperone-mediated autophagy (CMA) are two important subtypes of autophagy that play a critical role in cellular quality control under physiological and pathological conditions. Despite the marked differences between these two autophagic pathways, macroautophagy and CMA are intimately connected with each other during the autophagy-lysosomal degradation process, in particular, in the setting of neurological illness. Macroautophagy serves as a backup mechanism to removal of malfunctioning proteins (i.e., aberrant α-synuclein) from the cytoplasm when CMA is compromised, and vice versa. The molecular mechanisms underlying the conversation between macroautophagy and CMA are being clarified. Herein, we survey current overviews concentrating on the complex interactions between macroautophagy and CMA, and present therapeutic potentials through utilization and manipulation of macroautophagy-CMA crosstalk in the treatment of neurological diseases.

## Introduction

Autophagy is an evolutionarily conserved lysosomal degradation pathway by which cytoplasmic materials are delivered to and degraded in the lysosome [1]. It is essential for cell survival, differentiation, development, and homeostasis [2, 3]. Dysfunction of the autophagy-lysosomal pathway has been linked to a wide variety of brain pathology such as acute brain injuries [4, 5], neurodegenerative diseases (i.e., Alzheimer’s (AD), Parkinson’s (PD), and Huntington’s disease (HD)) [6–8], and brain tumors [9]. It is therefore significant to characterize the role of autophagy and its regulation in these diseases for therapeutic purpose.

Depending on the mode of cargo delivery to lysosome, autophagy can be subdivided into three subtypes: macroautophagy, microautophagy, and chaperone-mediated autophagy (CMA) [10]. Macroautophagy is the most universal form of autophagy characterized by the formation of autophagosome and is capable of disposing protein aggregates and damaged organelles [11, 12]. On the other hand, microautophagy is a lesser known self-eating event [13]. During the microautophagic process, cytosolic components are transported into the lysosome by direct invagination of lysosomal membrane and subsequent budding of vesicle into the lysosomal lumen [14]. In contrast, CMA is a unique pathway by which cytosolic protein aggregates are selectively transferred into lysosome for degradation [15]. Neither vesicle formation nor major changes in the lysosomal membrane occur during the CMA process [16]. Intriguingly, mounting evidences demonstrated that there is a functional relationship between macroautophagy and CMA, in particular, in the setting of neurological diseases [17, 18]. Dysfunction of either one of them can lead to a compensatory upregulation of the other. In this review, we aim to provide an integrative overview that concentrates on the interaction between macroautophagy and CMA in the mammalian systems (unless otherwise stated), and emphasize further details of the molecular mechanisms underlying the crosstalk between macroautophagy and CMA. Lastly, we will discuss therapeutic potentials by targeting macroautophagy-CMA crosstalk in the treatment of neurological diseases.

## Macroautophagy and CMA: Different Paths for Intracellular Protein Turnover and Recycling

### Macroautophagy: Process and Regulation

**Fig. 1:**
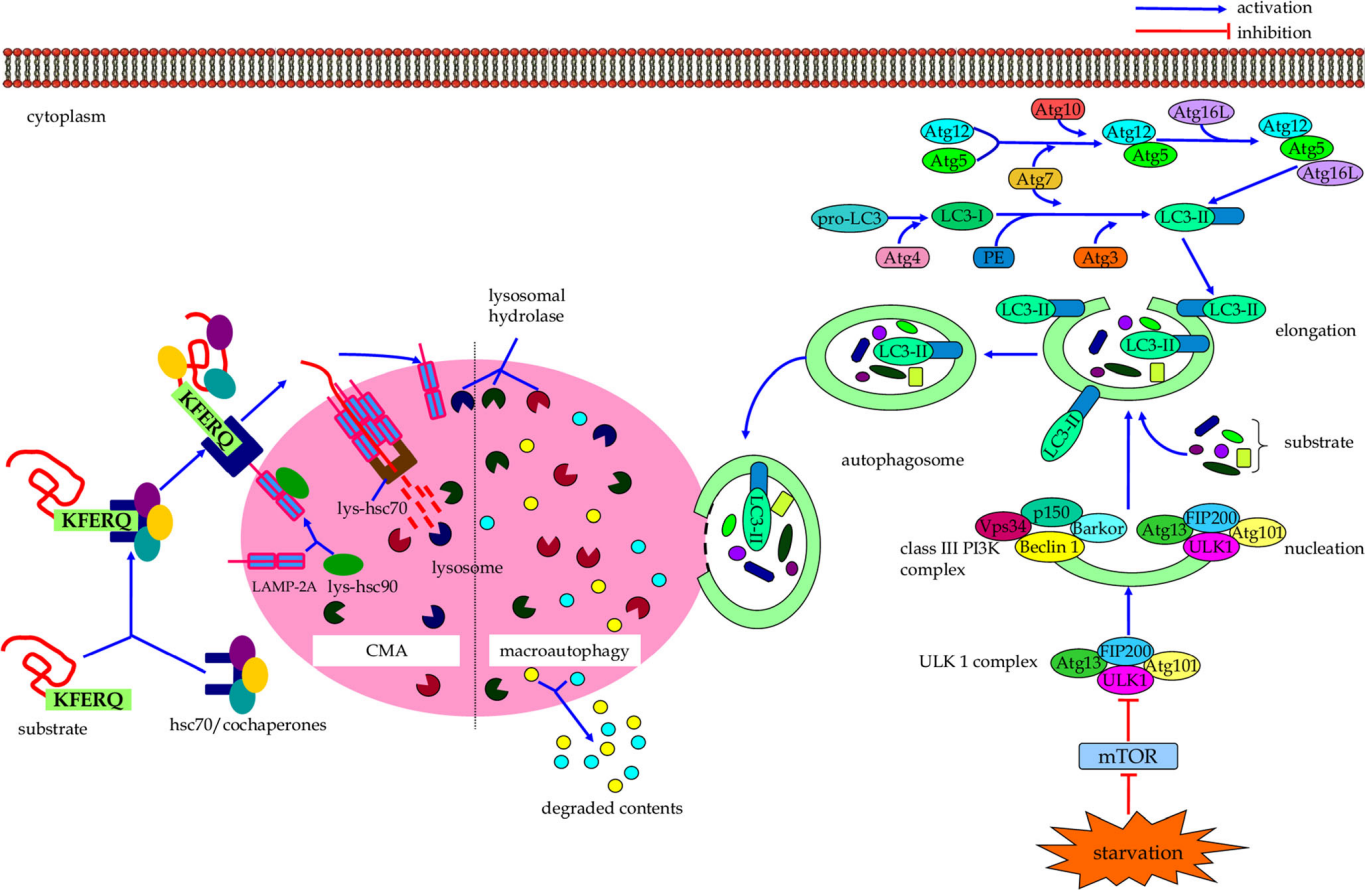
The cellular events of macroautophagy and CMA

The macroautophagic degradation of long-lived proteins and damaged/superfluous organelles consists of specific steps (Fig. 1) [19, 20]. It begins with nucleation of a unique membrane structure coined as phagophore, which is followed by the sequestration of cytoplasmic constituent into the phagophore. The elongation and closure of phagophore lead to generation of a double-membrane-bound autophagosome [21]. Autophagosome can fuse with lysosome to form autolysosome, where luminal contents of the autophagosome as well as inner membrane of the autophagic vacuole are broken down by lysosomal hydrolases [22]. The resultant biomolecules, such as amino acids, sugars, and lipids, are transported back into the cytosol for reuse via permeases functioning [23].

It is important to note that autophagy-related (Atg) proteins play an important role in autophagosome formation and macroautopahgy regulation [24–26]. For instance, the ULK1 complex, which consists of ULK1, Atg13, Atg101, and FIP200 (focal adhesion kinase family-interacting protein of 200 kD), is a vital complex in integrating incoming autophagy signals to initiate autophagosome biogenesis [27–29]. Beclin 1, the mammalian ortholog of yeast Atg6, can collaborate with other key subunits such as barkor (Beclin 1-associated autophagy-related key regulator), p150, and UVRAG (UV irradiation resistance-associated gene) to form the class III phosphatidylinositol 3-kinase (PI3K) complex that is crucial for nucleation and assembly of the initial phagophore membrane [30–33]. The elongation of pre-autophagosomal structures requires two ubiquitin-like modification systems, including Atg12-Atg5-Atg16L (Atg16-like protein) complex and LC3 (microtubule-associated protein 1 light chain 3) conjugation system [34–36]. In the first of these two ubiquitin-like conjugation reactions, the ubiquitin-like protein Atg12 is covalently tagged to Atg5 with the help of E1-like ubiquitin activating enzyme Atg7 and E2-like ubiquitin conjugating enzyme Atg10 [37–39]. The Atg12-Atg5 conjugate can then interact non-covalently with ATG16L to form the Atg12-Atg5-Atg16L tetramers, which function as a E3-like ubiquitin ligase that contributes to expansion of autophagosomal membrane by promoting LC3 lipidation [40, 41]. In the second ubiquitin-like conjugation reaction, LC3 is conjugated to phosphatidylethanolamine (PE) by E1-like Atg7 and E2-like Atg3 to form LC3-II [42, 43]. LC3-II can be specifically targeted to the elongating membrane, which aids the closure of autophagosomal membrane to form an autophagosome [44].

Multiple upstream signals, including mammalian target of rapamycin (mTOR)-dependent and -independent pathway, can regulate the process of macroautophagy [45, 46]. The serine/threonine protein kinase mTOR serves as a primary negative regulator of macroautophagy, and inactivation of mTOR promotes the macroautophagic process [47]. On the other hand, signaling pathways, such as cAMP-Epac-PLC-*ε*-IP3 pathway and Ca^2+^-calpain-G-stimulatory protein α pathway, can modulate macroautophagic response in an mTOR-independent manner [46, 48]. A more detailed regulatory mechanism of macroautophagy has been extensively described elsewhere [49–51].

### CMA: Process and Regulation

CMA is another subset of autophagy that has only been described in mammalian cells, and it is distinct from macroautophagy due to its specific mechanism of cargo selection and delivery to the lysosomal lumen for disposal [52]. In this form of autophagy, protein structures are selectively delivered into lysosome individually. Heat shock cognate chaperone of 70 kDa (hsc70) plays an important role in the CMA pathway (Fig. 1) [53]. Hsc70, together with its co-chaperones (e.g., hsc90), recognizes a specific consensus pentapeptide motif KFERQ in all cytosolic proteins targeting for CMA by forming a chaperone/substrate complex [54]. Initially, the chaperone/substrate complex is associated with lysosomal membrane protein receptor LAMP-2A (lysosome-associated membrane protein type 2A) [55]. The substrate protein can then be transported into lysosomal lumen for degradation with assistance of lysosomal-hsc70 (lys-hsc70) [56, 57].

The activity of CMA can be modulated by regulating local levels of LAMP-2A and lys-hsc70 at the lysosomal compartment [54]. Inhibition of p38 mitogen activated protein kinase (MAPK) can partially suppress the activation of CMA, suggesting the involvement of this signaling pathway in the modulation of CMA [58]. However, more signaling pathways which participate in the regulation of CMA remain to be clarified.

### Macroautophagy and CMA in Neurological Disorders

The autophagic-lysosomal systems, both macroautophagy and CMA, are important for cellular quality control to protect neurons from many kinds of damage and disease, such as acute injury, chronic neurodegeneration, and brain tumors. For instance, macroautophagy provides neuroprotective effects against ischemic, hemorrhagic, and traumatic brain injury [59–61]. Proper functioning of macroautophagy contributes to prevention of neurodegeneration via clearance of cytoplasmic aggregate-prone proteins and inclusions [62–64]. To date, the toxicity of causative gene products has been linked to the development of certain neurodegenerative disorder. Missense mutations in the amyloid precursor protein (APP) and presenilin genes can lead to an increase in the production of the amyloid-beta (Aβ) peptide, which can predispose to the development of AD-type brain pathology [65–69]. Pathogenic mutations in the tau gene can lead to abnormality in the function or isoform composition of this microtubule-associated protein, which contributes to the formation of abundant neurofibrillary lesions in AD and other tauopathies [70–73]. Stimulation of macroautophagy enhances the clearance of Aβ peptide and the APP-derived fragment in neurons and provides protective effects in cellular and animal models of AD [74–76]. Activation of macroautophagy can also eliminate both soluble mutant tau protein and its aggregates from the cytoplasm to promote neuronal survival in cellular and animal tauopathy models [77, 78]. PD is characterized by formation of neuronal intracellular Lewy body inclusions and the loss of dopaminergic neurons from the substantia nigra. Mutations in the α-synuclein gene (such as A53T and A30P) and the parkin gene are genetically linked to familial forms of PD [79–81]. In addition, mutations in the leucine-rich repeat kinase 2 gene (LRRK2) are clinically linked to PD [82–84]. These mutated gene products work together to promote the abnormal protein aggregation and Lewy body formation in PD [85–88]. More importantly, macroautophagy serves as a route for intracellular α-synuclein degradation to ameliorate the neurodegenerative pathology in PD models [89, 90]. In HD, an expansion of a CAG trinucleotide repeat in the interesting transcript 15 gene results to a mutant huntingtin protein with an abnormally expanded polyglutamine tract [91]. This cytotoxic polyglutamine-expanded huntingtin protein is highly expressed in neurons of the brain and can lead to the hallmark pathology of HD [92, 93]. Macroautophagy functions as a key clearance pathway for mutant huntingtin fragments that reduces intracellular huntingtin accumulation and protects cells against polyglutamine toxicity in HD models [45, 94, 63]. Similarly, activation of macroautophagy is beneficial for other so-called polyglutamine diseases, and it contributes to the degradation of mutant ataxin-3 protein that causes spinocerebellar ataxia type 3 [95–97]. Additionally, the mutant superoxide dismutase (SOD1) proteins, which are linked to familial amyotrophic lateral sclerosis, can be degraded by macroautophagy [98–100]. The impairment of the macroautophagy pathway is a crucial pathogenic mechanism of hereditary spastic paraplegia [101, 102]. Whereas, macroautophagy serves as a pro-death mechanism for brain tumor cells under certain conditions [103, 104]. Treatment with cytotoxic drugs such as arsenic trioxide and selenite triggers mitochondrial damage and initiates autophagic cell death in malignant glioma cells [103, 104].

Likewise, the signaling pathway of CMA is activated during hypoxic and ischemic stress to promote neuronal cell survival under these conditions [105]. Dysfunction of CMA causes accumulation of abnormal proteins in the pathological brain, which is involved in the pathogenesis of chronic neurodegeneration, such as PD [106], AD [107], and HD [108]. Indeed, the pathophysiological roles of CMA in the removal of aggregate-prone proteins that cause neurodegenerative diseases are being investigated. Evidence from both in vitro and in vivo studies supports that α-synuclein is a bona fide CMA substrate [109–111]. Wild-type α-synuclein, which contains a CMA-targeting motif in its sequences, can be delivered to lysosomes for its degradation via the CMA pathway [109]. Whereas, the pathogenic mutants of α-synuclein can bind to the CMA receptor LAMP-2A but fail to translocate inside the lysosomal lumen, thus impairing their own degradation along with that of other CMA substrates through this pathway [112]. Also, dopamine-modified α-synuclein can be bound to the lysosomal membrane, but it fails to translocate into lysosomes, resulting in the blockage of its degradation by CMA [113]. In the postmortem study of seven brain samples from PD patients, Alvarez-Erviti et al. reported a reduced expression of CMA proteins LAMP-2A and hsc70 in the substantia nigra pars compacta and amygdala of PD brains [114]. Downregulation of CMA activity compromises the degradation of alpha-synuclein, which underpins the Lewy body formation and PD pathogenesis [109, 115, 116]. Overexpression of LAMP-2A restores CMA activity and reduces the levels of aberrant α-synuclein and thereby ameliorates α-synuclein-induced dopaminergic neurodegeneration [106]. Although proteins like huntingtin contain CMA-targeting motifs in their sequences, the amount of these wild-type proteins degraded by CMA is negligible under normal conditions [117, 118]. However, in the case of HD, mutant huntingtin is selectively degraded by CMA through an hsc70 and LAMP-2A-dependent manner [108, 119]. Thus, functional regulation of this autophagy-lysosomal pathway provides novel means to prevent these devastating cerebral disorders.

### Crosstalk Between Macroautophagy and CMA: a Potential Target for the Treatment of Neurological Diseases

**Fig. 2:**
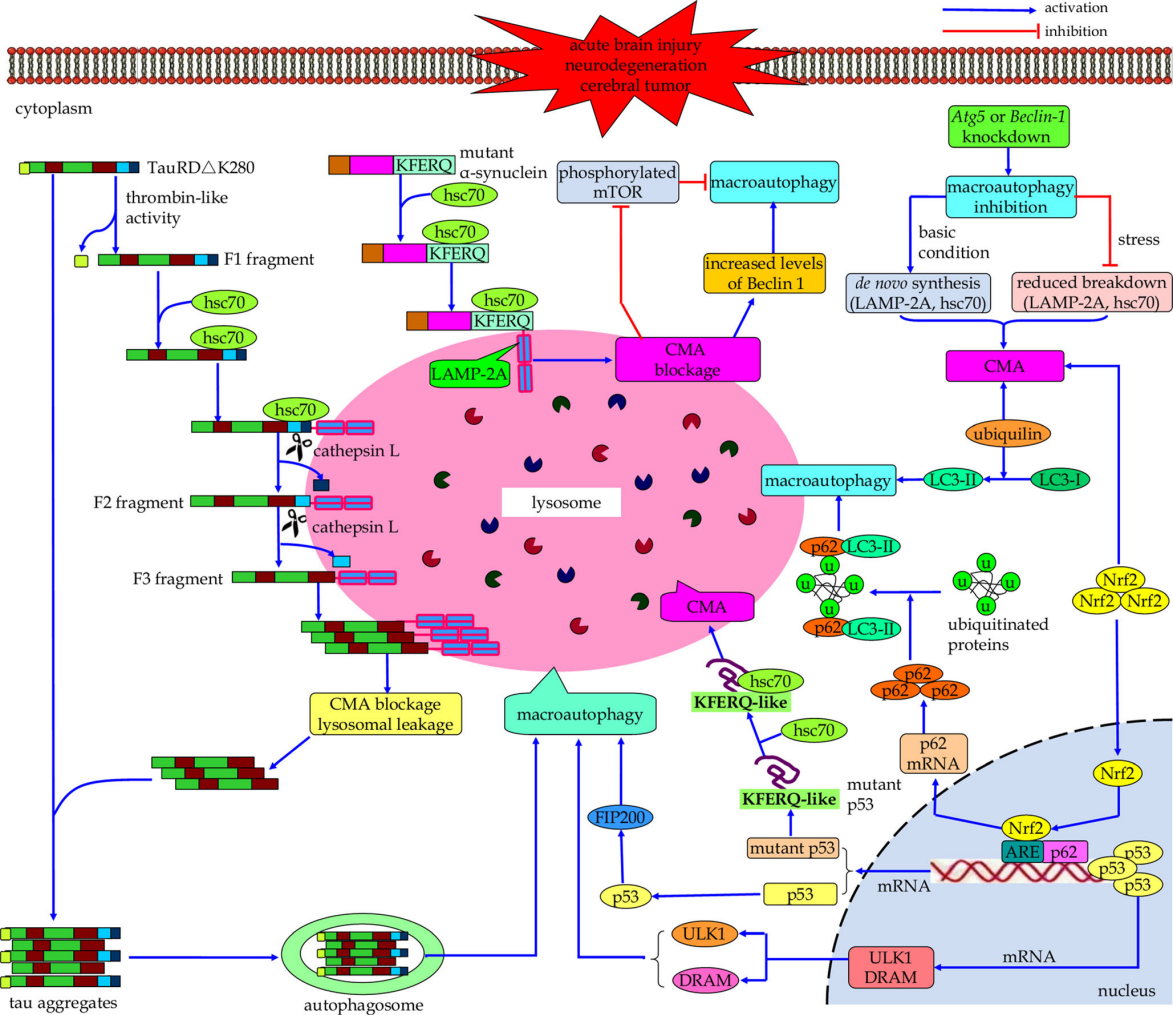
Schematic illustrating the interrelations between macroautophagy and CMA. Macroautophagy and CMA communicate with each other in the process of autophagy-lysosomal degradation. CMA blockage can induce compensative upregulation of macroautophagy, and the CMA pathway compensates when macroautophagy is inhibited. Molecular directors such as hsc70, Nrf2, ubiquilin, and p53 may function as core network nodes in macroautophagy-CMA crosstalk

Under normal cellular settings, macroautophagy and CMA occur at a basal level, but they can be activated when cells encounter stressful stimuli [54, 120]. During nutrient deprivation, the activity of macroautophagy rapidly upregulates and reaches the maximum level around 4∼6 h postfasting but downregulates immediately thereafter [121]. If the nutritional stress continues, the CMA pathway will be enhanced. The pathway activity peaks at 12∼20 h and remains to be active in the long-term starvation [122]. Increasing evidence has indicated that macroautophagy and CMA directly communicate with each other in protein degradation to maintaining cell homeostasis (Fig. 2) [123, 124]. For instance, in a cell model of tauopathy expressing TauRD△K280, Wang and colleagues proposed that the inability of complete degradation of mutated tau protein by the CMA pathway can lead to the pro-aggregating substrate generation and tau aggregation, resulting in tau pathology. At the same time, macroautophagy is able to clear these CMA-related tau aggregates, suggesting the functional interrelationships between macroautophagy and CMA [125, 117]. With series of key findings, molecular mechanisms underlying the crosstalk between macroautophagy and CMA are beginning to be elucidated. Dissecting these interactions between macroautophagy and CMA is essential for characterizing their respective roles in physiological and pathological conditions.

### CMA-Blockage-Induced Macroautophagy Upregulation

CMA is a cargo-specific subtype of autophagy which dictates multiple physiological functions [126]. It contributes to amino acid recycling during prolonged starvation [122] and fulfills protein quality control functions via selective turnover of damaged or malfunctioning proteins from the cytosol [127, 128]. As mentioned above, malfunction of CMA is implicated in the pathogenesis of cerebral diseases. In PD, the pathogenic A53T and A30P α-synuclein mutants can strongly bind to the CMA receptor LAMP-2A on lysosomes. However, these mutant forms of α-synuclein cannot be translocated into the lysosomal lumen, which inhibits not only the lysosomal uptake of their own but also that of other substrates for CMA degradation [109]. More importantly, in the PC12 cell model, pathogenic α-synuclein-mutant-mediated blockage of CMA leads to compensatory activation of macroautophagy, which makes up for portions of CMA’s functions in protein degradation [109]. In the cortical neurons and differentiated SH-SY5Y cells, the dysfunction of CMA conferred by mutant A53T α-synuclein can also result in an increased activity of macroautophagy [112]. However, under this condition, the compensatory upregulation of macroautophagy secondary to CMA inhibition due to overexpression of mutant A53T α-synuclein does not contribute to an increase of macroautophagy-dependent degradation. Instead, it causes the accumulation of autophagosomes that leads to membrane destabilization of these autophagic vacuoles and the cytoplasmic release of vacuolar hydrolases, thus eventually inducing autophagic cell death of primary cortical neurons [112, 129, 130]. The molecular mechanism underlying such a compensatory response of macroautophagy owing to CMA impairment, however, is still unclear. It has been demonstrated that in the early stage after CMA blockage by RNA interference against the lysosomal membrane receptor, LAMP-2A, its macroautophagic activity decreases because of an increase in mTOR activity. However, as CMA blockage persists, the increased intracellular levels of Beclin 1, along with the reduced phosphorylation of mTOR, can work together to induce constitutive activation of the macroautophagy[131].

Under basal conditions, compensatory macroautophagy is able to help CMA-defective cell to maintain normal protein degradation and cell viability [17]. However, upon exposure to certain stressors, such as pro-oxidative and oxidative stimuli (i.e., cadmium, paraquat, and H_2_O_2_), or to UV light, CMA-defective cell undergoes more apoptotic cell death. This suggests that despite the constitutive upregulation of macroautophagy, it is unable to compensate for all CMA functions [17]. It is proposed that the persistent activation of this pathway due to CMA blockage could lead to continuous consumption of the macroautophagic machinery proteins in chronically CMA-impaired cells and thus may limit a major increase in the activity of this pathway to respond to stress [131]. Therefore, in future research, it would be important to investigate whether constitutive activation of macroautophagy can always lead to the failure of its inducible form in response to stress, and to identify the time frame needed for this switch [131]. Additionally, considering the fact that aberrant activation of macroautophagy secondary to CMA inhibition leads to autophagic cell death of cortical neurons, improving the function of CMA function is important because it can mitigate potential deleterious consequences of aberrant induction of macroautophagy in this specific context of α-synuclein overexpression [112].

### CMA Compensates When Macroautophagy Is Inhibited

Macroautophagy is the bulk protein degradation pathway that plays an important role in protein quality control and maintaining cellular homeostasis of neural cells [8, 62]. A failure of macroautophagy-mediated clearance of misfolded proteins will cause the accumulation of these toxic proteins inside the affected neurons, leading to neurodegenerative disorders [132–134]. In the case of HD, activation of the kinase activity of inositol-requiring enzyme 1 mediates endoplasmic reticulum stress–induced inhibition of macroautophagic flux that causes accumulation of mutant huntingtin aggregates in cells, thus resulting in neuronal cell death [135, 136]. Martinez-Vicente et al. found that a defect in the ability of autophagic cargo recognition by autophagosomes compromises the removal of cytosolic components. As a result, there are increased levels of protein aggregates and abnormal intracellular lipid stores as well as a persistent level of dysfunctional mitochondria in HD cells [137]. Intriguingly, the impairment of macroautophagy can lead to activation of the CMA pathway under both basal and stress conditions [124]. An increase in the number of CMA translocation components (including LAMP-2A and hsc70) and CMA-competent lysosomes helps to boost the CMA activity following macroautophagy inhibition [124]. In cellular and mouse models, the HD cells are able to upregulate the activity of CMA pathway by increasing the de novo synthesis of LAMP-2A proteins and stabilizing of LAMP-2A at the lysosomal membrane followed by the blockage of macroautophagy in these cells [138]. It is proposed that cells obey different mechanisms to mediate the compensatory activation of CMA to respond to macroautophagy blockage depending on different conditions [127, 139]. The de novo synthesis of the receptor protein would be a major way to increase levels of LAMP-2A under basal conditions. In contrast, changes in the LAMP-2A protein already in the lysosomal compartment (i.e., reduced breakdown of LAMP-2A protein already at the lysosomal membrane) could be more optimal than de novo synthesis of this protein under stress conditions [124]. Therefore, it is interesting to determine whether or not there exist different mechanisms underlying compensatory upregulation of CMA secondary to macroautophagy blockage.

The compensative upregulation of CMA in response to macroautophagy inhibition promotes removal of oxidized proteins, which protects *Atg5* ^−/−^ murine embryonic fibroblasts from oxidative-stress-mediated cell death [140, 141]. Also, evidences from an in vivo study demonstrated that a compensatory CMA due to compromised macroautophagy provides a protective effect that contributes to the function preservation of cone retinal cells in conditional *Atg5* knockout in mice [142]. Notably, different with the evidence from *Atg5* null mouse embryonic fibroblasts [141], enhancement of CMA pathway secondary to macroautophagy inhibition fails to prevent RALA hepatocytes from menadione-induced oxidative stress and cell death [143]. These data suggest that macroautophagy and CMA have non-redundant functions in hepatocyte to resist to oxidant stress.

Taken together, macroautophagy is able to degrade the CMA substrates; however, it cannot compensate for selective degradation of particular substrates by CMA. Comparatively, CMA is capable of degrading cytosolic proteins but fails to take care of macroautophagy-mediated degradation of expired organelles. Despite these limitations, compensatory upregulation of one of them is beneficial for cells when the other is compromised, which helps cells to resist various types of damage to maintain cell viability [17, 141].

### Crosstalk Between Autophagy and Ubiquitin-Proteasome System (UPS)

The UPS is a major selective protein degradation pathway that plays important roles for eukaryotic cells [144]. UPS targets biologically non-useful proteins such as mutant, misfolded, damaged, or otherwise abnormal proteins for rapid substrate-specific proteolysis. In the CNS, UPS modulates a variety of basic cellular processes of neurons such as neuronal growth and development, synaptic function and plasticity, and neuronal survival and homeostasis [145]. Defect of the UPS has been linked to the pathogenesis of neurological disorders [146–148].

It is noteworthy that UPS and the autophagy-lysosomal system are functionally coupled in degrading excess or damaged proteins to maintain cellular homeostasis and neuronal survival [149, 150]. In a fly model of spinobulbar muscular atrophy, macroautophagy compensates for impaired UPS function in a histone deacetylase 6 (HDAC6)-dependent manner [151]. The compensatory macroautophagy due to proteasome inhibition plays an important role in controlling endoplasmic reticulum stress and reducing cell death [152, 153]. Also, in response to macroautophagy inhibition, proteasomes are activated in a compensatory manner for protein degradation [154]. Moreover, CMA and UPS collaborate to degrade the gene product of regulator of calcineurin 1, whose overexpression has been linked to Down syndrome (DS) and AD neuropathology [107]. Thus, further efforts are anticipated to explore the functional relationships between UPS and autophagy with the goal of controlling these interactions for optimizing the therapeutic approaches for cerebral diseases.

### Future Direction

Macroautophagy and CMA communicate with each other extensively in the autophagy-lysosomal degradation process. In certain circumstances, one can serve as a backup mechanism when the other is defective, and vice versa. However, research in the crosstalk between macroautophagy and CMA is still in its infancy. Better knowledge of the compensatory mechanisms between these two autophagic pathways, their advantages, and their limitations is of great significance, in order to develop effective interventions to regulate them for therapeutic purposes in autophagy-associated human disorders.

In particular, elucidation of the interrelationships between macroautophagy and CMA would have great clinical significance for intervention of those devastating cerebral disorders. As described above, macroautophagy and CMA are interconnected during protein degradation process in neurons. When either one is compromised, cells initiate the other autophagic pathway in a compensatory manner. However, inappropriate or excessive compensatory activation of autophagy may lead to neuronal cell death in the pathophysiological process of cerebral disorders. In addition, compensatory activation of the autophagic pathway may promote the development of brain tumors and its resistance to anticancer therapy [155–157]. Therefore, exploring the crosstalk between these two subtypes of autophagy helps to further define their exact roles in brain pathology (i.e., if they are the primary causes or the secondary consequences of pathological alterations). As a result, this will allow further molecular insights into the progressive dysfunction of autophagic protein degradation that leads to neurological diseases. It also provides key information for better manipulation of macroautophagy and CMA in the treatment and prevention of cerebral disorders.

Therefore, a series of critical issues regarding the macroautophagy-CMA network are warranted to be addressed. First, the physiological and pathological roles of macroautophagy and CMA require further investigation [126]. To date, little is known about the regulation of basal macroautophagy and CMA under normal conditions. Whether they function as a pro-death or pro-survival program in certain cerebral disorders should be addressed. Second, although there is evidence of interrelationships between macroautophagy and CMA [158], the temporal associations, the universality, the functional consequences, and the molecular directors of the macroautophagy-CMA chat are less understood. It is worthy to note that regulation of the levels of hsc70 in the lysosomal lumen during macroautophagy blockage offers clues about possible molecular mechanisms behind the crosstalk between these two autophagic pathways [124]. In macroautophagy-deficient cells, reduced fusion of autophagosomes with lysosomes could result in less dissipation of lysosomal pH. This causes more acidic environment in these lysosomes [124]. Lower lysosomal pH confers more stability of hsc70 in the lysosomal lumen to mediate CMA [124]. Recently, Rothenberg et al. demonstrated that ubiquilin proteins can be consumed by both macroautophagy and CMA [159]. Ubiquilin can positively regulate macroautophagy by promoting the conversion of LC3-I to LC3-II and accelerating autophagosome maturation [160]. However, it is actively degraded after carrying out its function during macroautophagic process. Moreover, ubiquilin contains two pentapeptide sequences (ILKDQ and EVRFQ) known as the CMA-targeting motif, which enable its selective recognition and degradation by CMA [159]. This dual mode of consumption of ubiquilin may have important implications in the crosstalk between macroautophagy and CMA. Therefore, it will be of interest to discover how ubiquilin is selected for either of these two forms of autophagy [159]. p53, the “guardian of the cellular genome” functions in the pathophysiology of cerebral diseases [161–163], may play a role in mediating macroautophagy-CMA crosstalk. On one hand, p53 can either promote or inhibit macroautophagy, depending on its subcellular locations [164–166]. On the other hand, inhibition of macroautophagy results in increased cytosolic levels of mutant p53 proteins in non-proliferating confluent cancer cells. This allows hsc70 to recognize their KFERQ-like sequence motifs and in turn enhances the degradation of mutant p53 in a CMA-dependent manner [18]. Additionally, the Nrf2 pathway could serve as an upstream signal to regulate the activities of both macroautophagy and CMA [167]. Genetical increase of astrocyte-specific Nrf2 can attenuate the functional deficiency of macroautophagy and CMA, which promotes the autophagy-lysosomal degradation of human mutant α-synuclein in the α-synuclein mutant mouse model [167]. Future investigations should focus more on key molecules such as ubiquilin, p53, and Nrf2, which might act as a core network node in the crosstalk between macroautophagy and CMA. Finally, large-scale proteomic- and metabolomic-based studies, as well as transgenic-based approaches, should provide valuable clues on the matter of macroautophagy-CMA crosstalk.

## Concluding Remarks

Macroautophagy and CMA exert critical roles in maintaining neuronal homeostasis in the brain under physiological and pathological conditions. Although considerable advances have been made in our insights into the macroautophagy-CMA network, key aspects regarding the interplay of these two catabolic pathways, including the compensatory mechanisms and their benefits and limitations, remain enigmatic. Answers to these questions are particularly significant to allow better manipulation of these two important autophagy-lysosomal pathways in the treatment of autophagy-related cerebral diseases.
